# A new generation of magnetoencephalography: Room temperature measurements using optically-pumped magnetometers

**DOI:** 10.1016/j.neuroimage.2017.01.034

**Published:** 2017-04-01

**Authors:** Elena Boto, Sofie S. Meyer, Vishal Shah, Orang Alem, Svenja Knappe, Peter Kruger, T. Mark Fromhold, Mark Lim, Paul M. Glover, Peter G. Morris, Richard Bowtell, Gareth R. Barnes, Matthew J. Brookes

**Affiliations:** a Sir Peter Mansfield Imaging Centre, School of Physics and Astronomy, University of Nottingham, University Park, Nottingham NG7 2RD, United Kingdom; b Wellcome Trust Centre for Neuroimaging, Institute of Neurology, University College London, 12 Queen Square, London WC1N 3BG, United Kingdom; c QuSpin Inc., 2011 Cherry Street, Unit 112, Louisville, CO 80027, USA; d Midlands Ultracold Atom Research Centre, School of Physics and Astronomy, University of Nottingham, University Park, Nottingham NG7 2RD, United Kingdom; e Chalk Studios Ltd., 14 Windsor Street, London N1 8QG, United Kingdom

## Abstract

Advances in the field of quantum sensing mean that magnetic field sensors, operating at room temperature, are now able to achieve sensitivity similar to that of cryogenically cooled devices (SQUIDs). This means that room temperature magnetoencephalography (MEG), with a greatly increased flexibility of sensor placement can now be considered. Further, these new sensors can be placed directly on the scalp surface giving, theoretically, a large increase in the magnitude of the measured signal. Here, we present recordings made using a single optically-pumped magnetometer (OPM) in combination with a 3D-printed head-cast designed to accurately locate and orient the sensor relative to brain anatomy. Since our OPM is configured as a magnetometer it is highly sensitive to environmental interference. However, we show that this problem can be ameliorated via the use of simultaneous reference sensor recordings. Using median nerve stimulation, we show that the OPM can detect both evoked (phase-locked) and induced (non-phase-locked oscillatory) changes when placed over sensory cortex, with signals ~4 times larger than equivalent SQUID measurements. Using source modelling, we show that our system allows localisation of the evoked response to somatosensory cortex. Further, source-space modelling shows that, with 13 sequential OPM measurements, source-space signal-to-noise ratio (SNR) is comparable to that from a 271-channel SQUID system. Our results highlight the opportunity presented by OPMs to generate uncooled, potentially low-cost, high SNR MEG systems.

## Introduction

Magnetoencephalography (MEG) is a non-invasive technique for imaging electrophysiological brain activity (Cohen, 1972). Dendritic current flow, synchronised across neural assemblies, generates small changes in magnetic field outside the head. An array of highly sensitive magnetic field sensors is used to detect these small changes and 3-dimensional images depicting moment-to-moment changes in brain current are then reconstructed using mathematical modelling. In the past decade, MEG has become an important tool for neuroscience providing a bridge between the spatially detailed (but slow and indirect) haemodynamic imaging measures in humans, and the temporally rich (but sparsely spatially sampled) information provided by invasive electrophysiological metrics in animals (Hall et al., 2005, Zumer et al., 2010). MEG is capable of tracking the formation and dissolution of electrophysiological networks in real time and with high spatial precision (O'Neill et al., 2015, Troebinger et al., 2014a). Because of this, MEG is now having impact on our understanding of the healthy brain, and on a wide range of clinical research areas ranging from neurodevelopment (Ciesielski and Stephen, 2014) to severe psychoses (Robson et al., 2016, Uhlhaas and Singer, 2010). The further development of MEG technology is therefore an important and evolving focus.

Despite its potential, MEG is limited by low signal-to-noise ratio (SNR). This is because the magnetic fields generated by the brain are much smaller than environmental and biological magnetic interference. This problem severely limits sensitivity (e.g. comparisons of invasive local field potential metrics (Mukamel et al., 2005) and MEG (Zumer et al., 2010) suggest that the latter is relatively insensitive to networks oscillating at high frequency). Moreover, many studies have shown how improvements in SNR give direct benefits in terms of spatial resolution in MEG (Brookes et al., 2008, Brookes et al., 2010, Gross et al., 2003, Hillebrand and Barnes, 2002, Troebinger et al., 2014a, Vrba, 2002). In principle, SNR could be improved by moving detectors closer to the head; this is because the field from the brain follows an inverse square law meaning that halving the source-to-detector separation would quadruple the measured signal amplitude. However, current MEG systems are based around superconducting circuits housed in a liquid-helium-cooled dewar. This means that thermal insulation must be maintained between the scalp and detector, and so detectors are sited 3–6 cm from the adult brain surface. The requirement for cryogenic cooling also means that sensors are held in a fixed position within a ‘one size fits all’ helmet. The average brain-to-sensor distance is therefore even greater in subjects with smaller heads (e.g. infants). Further, the inflexibility in sensor placement means that some regions can have poor coverage (e.g. the cerebellum). It follows that the introduction of room-temperature detectors, with a similar performance to superconducting devices, but enhanced flexibility to enable positioning anywhere on the scalp surface, would bring about a step change in the capability of MEG. Indeed simulations (Boto et al., 2016) have shown that a MEG system with a similar geometry to current instruments, but with scalp-mounted detectors, would afford approximately a fivefold improvement in signal magnitude (depending on subject head size and location of interest in the brain), and a similar improvement in source-space SNR and spatial resolution.

Recent advances in the field of quantum technology have led to the development of small, optically-pumped magnetometers (OPMs). These devices measure the transmission of laser light through a vapour of spin-polarised rubidium atoms, which provides a highly sensitive measure of the local magnetic field. OPMs have theoretical sensitivity comparable to that of the superconducting quantum interference devices (SQUIDs) used in current MEG systems (Dang et al., 2010), but operate without cryogenic cooling (the vapour cell is heated, but the external surface remains close to room temperature). This means it is now possible to consider a MEG system with sensitive detector volumes just 6 mm from the scalp surface. Robust and easy to use OPM sensors have recently become available commercially; these sensors are fabricated such that they have a small footprint meaning that a large number of sensors can be placed flexibly around the head and whole-head coverage is feasible. The utility of OPMs for MEG has been shown previously: several papers have described the successful detection of phase-locked evoked responses generated by auditory (Johnson et al., 2010, Johnson et al., 2013, Xia et al., 2006) or somatosensory (Johnson et al., 2010, Sander et al., 2012) stimulation. Other groups have shown how sensors positioned over the occipital lobe enabled detection of the induced changes in alpha (8–13 Hz) rhythm by opening and closing the eyes (Kamada et al., 2015, Sander et al., 2012). In addition, a recent study has demonstrated the detection of epileptiform activity in rodents (Alem et al., 2014). These empirical demonstrations, coupled with recent commercialisation, decreased sensor size (Shah and Wakai, 2013) and the introduction of multi-channel measurements (Colombo et al., 2016, Johnson et al., 2013, Kim et al., 2014) show the potential of OPMs to transform the utility of MEG, with the promise not only of high SNR, but also access to traditionally challenging subject groups such as infants.

Although OPMs have the potential to form the basis of a high SNR and flexible MEG instrument, the practical implementation of such a system requires significant work. The increased signal provided by moving the detectors closer to the scalp is easily achieved. However translating this into high precision images of electrical brain function is non-trivial. First, most OPMs are configured as magnetometers (as distinct from gradiometers which are used in many SQUID-based MEG systems) and as such are more sensitive to environmental interference. Effective methods must therefore be employed to reduce interference. In addition, spatial specificity and reconstruction accuracy depend not only on SNR, but also on accurate modelling of magnetic fields generated by the brain (i.e. accurate forward solutions). This problem is exacerbated in OPMs since, perhaps counterintuitively, increases in SNR mean that modelling accuracy must also increase; indeed simulations show that even small (5%) modelling errors could negate any advantages in SNR afforded by OPM systems (Boto et al., 2016). One significant cause of modelling errors in current MEG systems comes from inaccurate knowledge of the head location relative to the sensor array (Hillebrand and Barnes, 2003). It follows that to realise the benefits of an OPM system, detector locations and orientations relative to the brain anatomy must be known with high spatial accuracy. In this paper, we show that the use of a 3D-printed head-cast can accurately inform sensor position and orientation, thus facilitating accurate forward modelling and source imaging. We employ a single commercial OPM (QuSpin Inc., Louisville, CO, USA) to make measurements of evoked (phase locked) and induced (non-phase locked oscillatory) neural signals in sensory cortex during median nerve stimulation. We show that the use of interference cancellation via a reference array, i.e. similar to ideas behind synthetic gradiometers (Vrba, 2002, Vrba et al., 1991, Vrba and Robinson, 2002, Vrba and Robinson, 2001) improves significantly the SNR of OPM measurements. Finally, we show that OPMs generate the expected increases in signal magnitude over SQUID recordings.

## Methods

### Data acquisition

#### Optically-pumped magnetometer

The OPM used in this work has a theoretical noise level comparable to SQUIDs (10 fT/√Hz above 1 Hz), a bandwidth greater than 100 Hz, an operational dynamic range of ±5 nT, a size of 14×21×80 mm^3^, and can be placed such that the sensitive volume is 6.5 mm from the scalp head surface. The OPM sensor head is self-contained with all the necessary optical components, including a semiconductor laser for optical pumping, optics for laser beam conditioning, a 3×3×3 mm^3 87^Rb vapour cell, and silicon photodiodes. The vapour cell is electrically heated to around 150 °C to achieve optimum ^87^Rb vapour density. The sensor head connects to a small electronics controller via a 5 m cable. The electronics controller is placed outside the magnetically-shielded room (MSR) to minimize magnetic interference. The output from the electronics controller is an analogue voltage proportional to magnetic field, which is digitized using a 16-bit DAQ system. The effective resolution of this digitisation is increased via oversampling at 10 kHz. The scaling of output voltage to measured field is 2.8 V/nT.

**Fig. 1:**
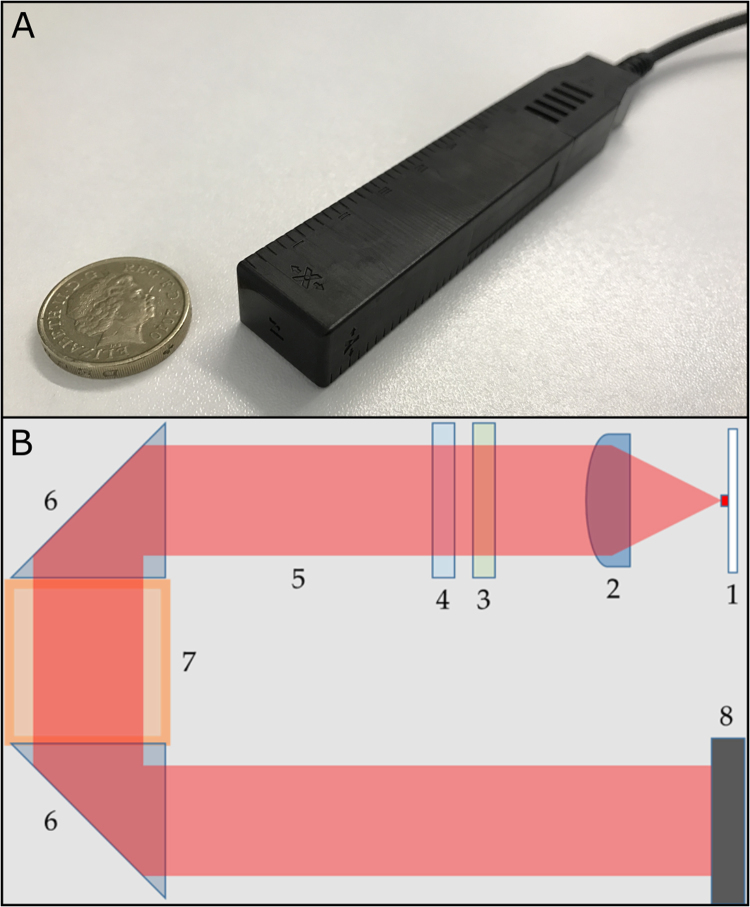
**The OPM sensor**. A) The QuSpin OPM sensor, next to a UK pound coin, which has a semiconductor laser and gas cell mounted in a housing of 14×21×80 mm^3^. The field sensing area is at 6.5 mm from the end of the housing. B) Schematic showing the basic operation. 1: 795 nm laser. 2: Collimating lens. 3: Linear polariser. 4: Circular polariser. 5: Light beam. 6: Reflecting prisms. 7: Vapour cell. 8: Photodiode. (Coils not shown). The amplitude of the two components of the magnetic field that are perpendicular to the beam can be simultaneously measured via assessment of changes in light intensity at the photo-diode.

The magnetometer relies on a zero-field level crossing resonance for detection of ambient magnetic field, described in detail by Kastler (1973) and Dupont-Roc et al. (1969). A 795 nm laser tuned to the D1 transition of ^87^Rb is used to spin-polarize the rubidium atoms, and the intensity of laser light transmitted through the cell is detected using a photodiode. The sensor includes three electromagnetic coils which can be used to null any static field components in the cell; subsequent field changes (e.g. due to neural currents in the brain) can then be detected via the change in transmitted light intensity which they produce. The transmitted intensity manifests a zero-field resonance, which is a Lorentzian function of the magnetic field components transverse to the laser beam, with a full width at half maximum of 30 nT. A small, sinusoidally modulated magnetic field is applied at ~1 kHz perpendicular to the laser beam by the on-sensor coils. The modulation of the transmitted light, which is monitored using a lock-in process, is sensitive to the ambient field component along the modulation axis (Dupont-Roc et al., 1969, Shah et al., 2007). The amplitude of the two components of the magnetic field that are perpendicular to the beam can be simultaneously measured by applying oscillating currents to two coils in quadrature. A photograph of the sensor is shown in Fig. 1A. Fig. 1B shows a basic schematic illustrating the operational principle. Note here that although the sensor is sensitive to both radial and transverse oriented magnetic fields, only the radial field component was measured.

#### Head-cast design

**Fig. 2:**
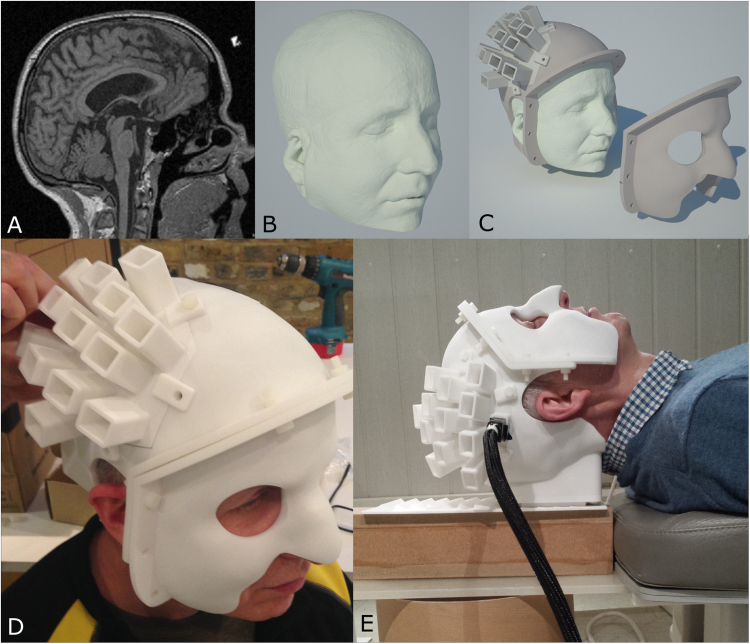
**Head-cast design and fabrication:** A) Single sagittal slice from the anatomical MRI. B) Outer head surface extracted from MRI. C) CAD model of the head-cast with slots designed to house the OPM sensors over sensorimotor cortex. Slot positions are based on a-priori prediction of the spatial topography of scalp field pattern, derived from previous SQUID measurements in the same subject. D) 3D-printed head-cast on subject. E) Subject in situ with the OPM attached. Note that the head-cast is not only fixed rigidly to the subject's scalp, but is also fixed relative to the MSR, thus eliminating any sensor motion relative to the subject, and subject motion relative to the MSR.

There are two practical problems with using a single OPM to detect neuromagnetic fields: first, the convoluted shape of the cortex means that, even if the approximate location of the region of interest in the subject's head (somatosensory cortex in our case) is known, the precise orientation of the local normal to the cortical surface is unknown. Since the locations of field maxima outside the head are strongly dependent on the direction of current flow, this means that it is difficult to predict where on the scalp sensors should be optimally placed. Second, in order to mathematically model the measured fields, and hence derive 3D images of changing cortical current flow, the precise location and orientation of the sensors relative to the brain anatomy must be known. We solved both problems via the use of 3D printing (see Fig. 2). We used an anatomical magnetic resonance image (MRI) of the subject's head (as described in Meyer et al. (2017)) (Fig. 2A) in order to extract a 3D mesh, representing the outer surface of the head and face (Fig. 2B). Following this, a nylon cast of the outer head surface was fabricated using 3D printing (http://www.chalkstudios.co.uk/), resulting in a head-cast that is moulded to the shape of the individual subject's head (Fig. 2C/D). As part of the head-cast design, we digitally placed a nominally hexagonal array of slots for 13 radially-oriented OPMs over the subject's right somatosensory cortex. The array was located and oriented so as to sample the field maxima and minima expected at 6.5 mm from the scalp surface due to a dipole in the somatosensory cortex (whose location and orientation was estimated from previous SQUID-based dipole fits to the same subject). Importantly, since the head-cast was generated directly from the subject's MRI, the precise location and orientation of the slots for the sensors, with respect to the brain anatomy, was known. The multiple slots meant that the single OPM could be placed in any one of the 13 locations in order to sample the spatially variation of scalp-level magnetic fields.

#### Median nerve stimulation and data collection

The somatosensory paradigm involved electrical stimulation of the subject's left median nerve, which was achieved by applying a series of 500-μs-duration current pulses to two gold electrodes placed on the subject's wrist. The current was delivered using a Digitimer DS7A constant current stimulator, and the amplitude was increased until a visible movement of the thumb was observed. Each experimental run comprised 80 pulses delivered with an inter-stimulus-interval (ISI) of 3.0099 s; the ISI was selected not to be a multiple of the 20 ms period of the mains noise frequency (50 Hz). Data were recorded during a pre-stimulus baseline of 0.5 s and post stimulus period of 2 s. A single run lasted four minutes. All recordings were carried out inside the MSR comprising two layers of mu-metal and one of aluminium. During recording, the lights in the control suite (which operate at 50 Hz) were turned off, as was any unnecessary equipment, to minimize ambient interference. Lights in the MSR were also off.

##### SQUID data

The experiment was carried out using a 275-channel whole-head CTF MEG system (MISL, Port Coquitlam, Canada) operating in third-order synthetic gradiometer configuration. The subject was positioned supine, with their head in the MEG helmet, whilst data were recorded at a 600 Hz sampling rate. Three coils were attached to the participant's head as fiducial markers at the nasion, left and right preauricular points. These coils were energised periodically throughout acquisition to allow localisation of the head relative to the MEG sensor array. Before acquisition, the surface of the participant's head was digitised using a 3D digitiser (Polhemus Inc.). Subsequent surface matching of the digitised head shape to an equivalent head shape extracted from the anatomical MRI allowed co-registration of brain anatomy to MEG sensor geometry. Note that the CTF system comprises 275, 5 cm baseline axial gradiometers spaced evenly across the scalp surface, which are magnetically coupled to 275 SQUID sensors. Here, to simplify terminology, we refer to these as SQUID sensors throughout the manuscript.

##### OPM data

Prior to the SQUID measurement, the median nerve experiment was carried out 13 times and MEG data recorded using a single OPM. The subject was positioned on the bed of the CTF system, with their head inside the head-cast and the head-cast fixed to the bed (Fig. 2). On each repeat of the experiment, the OPM was positioned in a different slot in the head-cast. In this way, we simulated a multi-channel MEG recording. The analogue output of the OPM was captured by analogue-to-digital converters on the CTF MEG system (16 bit sampling with oversampling to maximise resolution). This allowed simultaneous data capture from the OPM as well as all channels in the CTF system. All channels, including the OPM were sampled at 600 Hz. Note that the CTF system also incorporates 29 reference channels, located in a separate array, distal to the subject's head. We exploit the simultaneous capture of OPM and SQUID data to compensate for interference. In addition to the median nerve experiment, 100 s of data were also recorded with no subject present, to assess ambient noise levels.

### Data analysis

Initially, all MEG data (from the SQUIDs and the OPM) were filtered, either between 2 Hz and 80 Hz (empty room recording) or 0 and 150 Hz (median nerve data – the 2 Hz high pass filtering was omitted due to concerns regarding filter artefacts). For the median nerve data the DC offset was removed based on the 500 ms pre-stimulus period. There was a delay between the SQUID and OPM recordings, with the SQUIDs leading the OPM by 3 data points (5 ms). This was caused by the digital signal processor running the OPM, and was measured by assessing the phase of the 50 Hz mains artefact measured using the OPM compared with the reference SQUID magnetometers. Having characterised the delay, it was corrected by time-shifting the SQUID measurements. All data analyses were undertaken using Matlab (Mathworks Inc.).

#### Synthesised gradiometer formulation

In the current generation of commercial MEG devices, interference is compensated by the use of both hardwired gradiometers and reference arrays (e.g., used to form higher-order gradients; Vrba (2000)). However, our single OPM is configured as a magnetometer with no compensation for interference. Here, we use the simultaneous recording of OPM and SQUID data to create a synthesised software gradiometer. SQUID sensor locations (which were between 50 cm and 100 cm from the OPM slots) were close enough to be sensitive to similar environmental interference, but far enough away that they were not sensitive to neuromagnetic signals. The CTF instrument was therefore used as a reference array; data were augmented by using the differentials with respect to time of the magnetometer channels, so as to allow any linear mixtures to have variable phase. Data were then combined in a single (design) matrix and a linear regression was used to remove any linear combination of SQUID (interference) signals from the OPM. In the median nerve data, this regression applied trial by trial, and in the 0–150 Hz range. For empty room data, the regression was applied over the full 100 s recording (since there were no trials). We also contrasted its application in the full 2–80 Hz frequency range, and independently within separate (overlapping, 5 Hz) frequency bands.

#### Evoked and Induced responses

After correcting for interference using synthesised gradiometry, we assessed the sensor level evoked and induced signals from the OPM and SQUIDs by forming the average over trials. To compare the temporal morphologies and response magnitudes of SQUIDs and OPMs, the SQUID signal exhibiting the largest evoked response was taken and compared with the OPM with the largest evoked response. In addition to the averaged response, unaveraged evoked responses were also extracted from the OPM data (with and without synthetic gradiometer correction) to examine single trial measurements.

A time-frequency analysis was used to evaluate the induced (oscillatory) response. Sensor time-series were frequency-filtered into a set of overlapping frequency bands and, for each frequency band, a Hilbert transform was used to calculate the analytic signal. The absolute value of the analytic signal was then derived to generate the amplitude envelope of oscillations (termed the Hilbert envelope). These Hilbert envelope time courses were averaged across trials and concatenated in the frequency dimension to generate a time-frequency spectrum (TFS). The SQUID/OPM locations with the largest response were used.

#### Source localisation and signal-to-noise ratio

We used inverse modelling to assess whether or not we could use OPM measurements to reconstruct the cortical generator of the evoked (N20) response. In doing so, we used the recordings made with the OPM sensor at different positions in the head-cast to simulate a multichannel array, analysing all the data as if recorded simultaneously. Following the noise cancellation procedures, we baseline-corrected each sensor to the −500 ms to −100 ms time window relative to stimulus onset. This allowed us to identify the temporal peak of the N20 response. We used a single shell model of the inner skull surface and a Bayesian dipole fitting procedure (similar to Kiebel et al. (2008)), implemented in SPM12 (http://www.fil.ion.ucl.ac.uk/spm) to explain the OPM measured fields at the time of the peak N20 response. This Bayesian fitting scheme assigns a prior mean and precision to the dipole location and moment parameters. We randomly chose 20 starting points (as the prior mean locations) within the brain and assigned an uninformative prior spatial (standard deviation of 10 cm) to each of these locations. The prior mean and standard deviation of each moment parameter was set to 0 and 10 nA· m respectively. The posterior location and moment parameters were optimized for each set of priors, so as to maximize the model evidence (or Free energy) over ten iterations (each iteration having a random starting point drawn from the prior distributions). Of these models and iterations, the most likely solution was taken to be the one with the highest overall model evidence. This same methodology was also applied to the SQUID recordings for comparison.

A direct comparison of sensor-space SNR using OPMs and SQUIDs is confounded for two reasons: first, it involves comparison of signals measured using a SQUID gradiometer and OPM magnetometer. Second, significant ‘interference’ in both systems comes from brain sources of no interest. By moving a detector closer to the brain (i.e. using an OPM rather than the SQUID), the sensitive brain volume (contributing the sources of no interest) changes (see Appendix A). Specifically, a greater proportional sensitivity to shallow, compared to deep, sources is observed. This changing volumetric sensitivity profile means that the OPM and SQUID sensors have fundamentally different interference generators. For these reasons, the only means to generate unbiased estimates of SNR is to reconstruct source-space signals. Here, the dipole time course was reconstructed using a linear weighted sum of sensor measurements; weights were derived using our dipole fitting procedure. This was done independently for the 13 OPM measurements, and the 271 SQUID measurements (see also Supplementary information). SNR was measured in two ways: in both cases signal was estimated as the peak-to-peak change in the evoked response. In the first case, noise was estimated as the standard deviation in a baseline window between 0.6 s and 1.5 s post stimulus. In the second case, noise was estimated using a procedure whereby alternate trials were sign flipped prior to averaging. Following this anti-averaging procedure, the standard deviation of the whole trial was used to estimate noise.

## Results

**Fig. 3:**
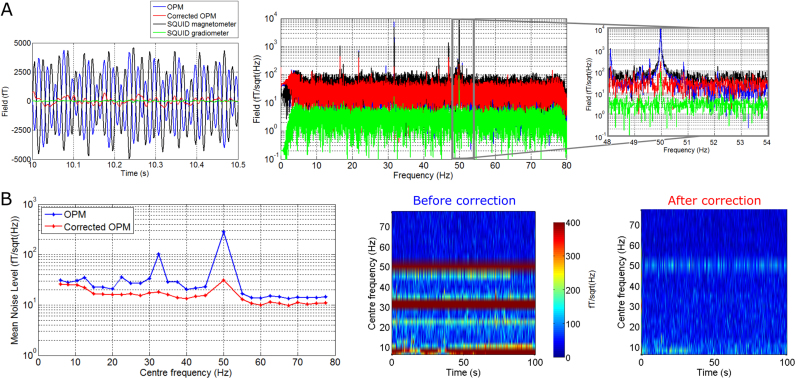
**Synthesised Gradiometry (empty room noise).** A) Magnetic fields plotted in time (left) and frequency (right), measured in an empty room. Red and blue traces show the OPM data with and without gradiometer correction respectively. The black trace shows a SQUID magnetometer and the green trace shows a SQUID gradiometer. Note that 97% of variance in the OPM signal was explained by the simultaneously measured SQUID reference sensors and their temporal derivatives. Here, gradiometry was applied to the full (100 s) time window and full (2–80 Hz) bandwidth. B) Left hand side shows a noise amplitude spectrum when gradiometer correction is applied to individual frequency bands. Uncorrected (blue) and corrected (red) OPM mean noise levels across 28 bands are shown. Centre and right panels show time-frequency spectra in fT/√Hz, before and after gradiometer correction.

Fig. 3 shows results of applying the synthetic gradiometer to the OPM data recorded in an empty room. Fig. 3A shows magnetic field plotted as a function of time (left hand plot) and in Fourier space as a function of frequency (right hand plot). The red and blue traces represent OPM measurements with and without synthetic gradiometer correction respectively. For comparison, the black trace shows a SQUID magnetometer (selected randomly from the reference array) and the green trace shows a SQUID gradiometer. Note that 97% of the variance measured at the OPM could be explained by interference measured simultaneously the SQUID array. In the Fourier plots, the centre panel shows the full frequency range, whilst the right hand panel shows data around the 50 Hz peak. The uncorrected OPM has a mean noise level (MNL) of 31 fT/√Hz in the 2–80 Hz frequency range, which reduces to 26.2 fT/√Hz when using synthetic gradiometry. However this reduction is clearly dominated by the 50 Hz mains frequency peak (which reduces from 12,512 fT/√Hz to 180 fT/√Hz) and a 32 Hz peak (which reduces from 7,642 fT/√Hz to 314 fT/√Hz). Whilst the origin of the 32 Hz peak is unclear, it was thought to relate to site-specific interference from an air conditioning system. The gradiometer formulation used here is simple; only a single (regression) mapping is employed to remove interference measured at the reference array from the OPM, meaning the necessary assumption that all interference sources have the same spatial profile. The mapping is thus dominated by the largest interference source (50 Hz) which explains why this gradiometer formulation is efficient in removing mains interference, but has little effect at other frequencies. By comparison, Fig. 3B shows gradiometer correction applied to individual frequency bands (5 Hz overlapping bands between 2 and 80 Hz). Here, we assume a different gradiometer mapping for each band (i.e. regression coefficients are derived optimally for all bands, allowing interference sources in different bands to exhibit different spatial profiles). The left panel shows the uncorrected (blue) and corrected (red) noise levels across 28 bands. The centre and right panels show time-frequency spectra before and after correction. Note that, as well as a marked reduction at 50 Hz, the efficiency of the synthesised gradiometer at other frequencies is also improved with mean interference (across bands) falling from 34.5 fT/√Hz pre-correction to 15.9 fT/√Hz post-correction.

**Fig. 4:**
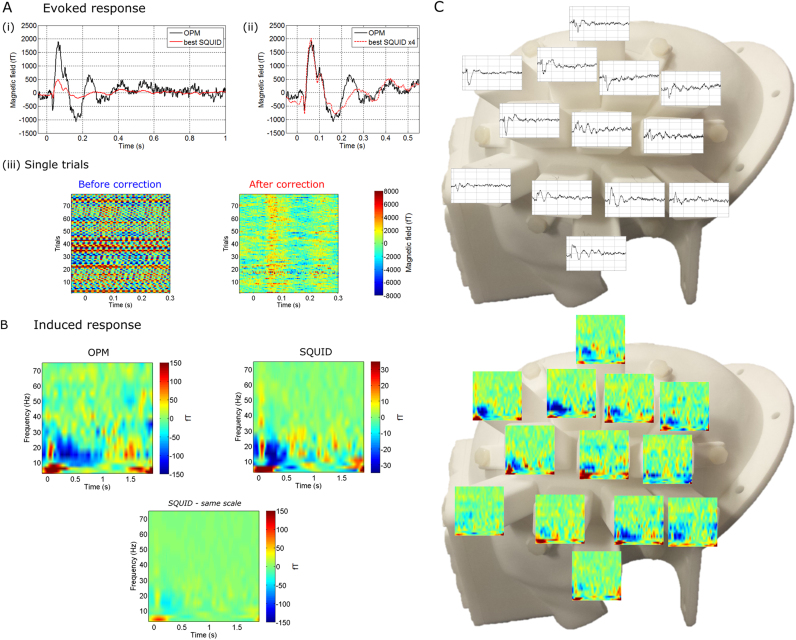
**Comparison of the magnitudes and (spatial, spectral and temporal) morphologies of the evoked and induced responses, measured using the OPM and the SQUID**. A) (i) Measurements of the evoked response (in fT) for both the OPM (black) and SQUID (red). (ii) shows the same data as in (i) but with the SQUID time course multiplied by four to allow direct comparison of temporal morphology. (iii) Single trial evoked responses measured using OPM with (right) and without (left) gradiometer correction. Note that single trial responses are seen clearly. B) Time-frequency spectrograms showing the 0–80 Hz oscillatory signature of median nerve stimulation. Upper left hand panel shows OPM, upper right hand panel shows SQUID, and lower panel shows SQUID plotted on the same colour scale as the OPM, for comparison. C) The scalp level spatial topographies of the evoked and induced responses, overlaid onto the head-cast.

Fig. 4 shows a comparison of evoked and induced responses measured at the sensor level using the OPM and the SQUIDs. Fig. 4A(i) shows the absolute magnitude of evoked responses (measured in fT) for the OPM (black) and the SQUID (red). In both cases, responses from the sensor location yielding the maximum response magnitude are shown. The close proximity of the OPM to the scalp surface produces approximately a fourfold increase in the magnitude of the measured signal; in line with previous simulations (Boto et al., 2016). Fig. 4A(ii) shows the same signals, but the SQUID signal is multiplied by four such that temporal morphology can be compared; note the similarity in the OPM and SQUID measured responses. Fig. 4A(iii) shows single trial OPM responses before and after gradiometer correction. Note here that the x-axis represents time, the y-axis represents trial number, and the colour represents measured field. The removal of interference (dominated by reduction in 50 Hz mains artefact) allows single trial evoked responses to be seen following correction. Fig. 4B shows time-frequency spectrograms representing the induced response to median nerve stimulation. It is well known that, in the sensorimotor system, movement and sensory stimulation is linked to changes in neural oscillations in the beta band (see Cheyne (2013) and Kilavik et al. (2013) for reviews). Specifically, following stimulation, a decrease in the amplitude of beta oscillations is observed followed immediately by a period of elevated amplitude, known as the post-stimulus beta rebound. This bimodal amplitude change, which occurs simultaneously with the evoked response, has been well characterised in previous work, and is extremely robust across individuals. Here, using both the SQUID and OPM, we observed the expected modulation with a stimulus-induced decrease and rebound in beta amplitude relative to baseline. Note again the similarity of time-frequency morphology of the response, and the approximate fourfold difference in scale of the two measurements. The left hand plot shows the TFS for an OPM, the upper right plot shows the case for the SQUID, and the lower plot shows the SQUID measurement plotted on the same colour scale (−150 fT to 150 fT) as the OPM. Fig. 4C shows the scalp-level spatial topographies of both the evoked and induced responses. Both are overlaid onto a photograph of the 3D-printed head-cast.

**Fig. 5:**
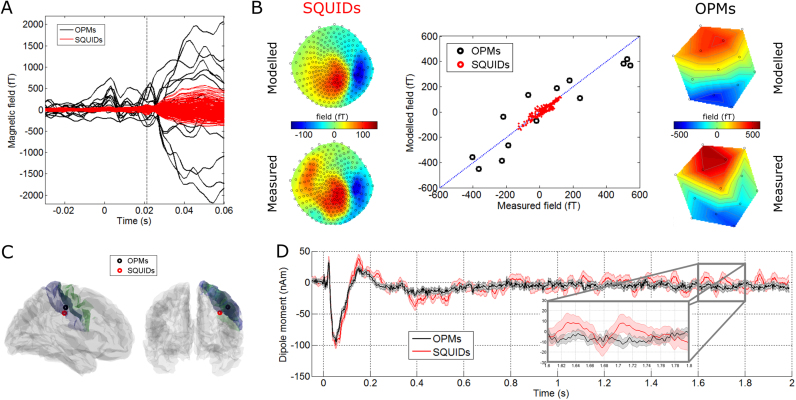
**Source localisation of the evoked response:** A) Averaged time courses of OPM data (black) recorded at the 13 different sensor locations across the head-cast, and SQUID data (red) corresponding to the 271 SQUID channels. Note the electrical stimulus artefact at t=0 and the evoked response peak at t=20 ms. B) Left and right panels show the scalp spatial topography (for the SQUIDs and OPMs, respectively) of the measured (bottom) and modelled (top) fields. The centre plot shows correlation between the modelled and measured fields for the OPMs (black) and SQUIDs (red). C) Two views of the N20 evoked response location. Note that the motor strip is shown in green and the sensory strip in blue. The peak localises to right primary sensory (S1) region as expected. The black marker shows localisation using the OPM system and the red marker shows equivalent localisation using the SQUID system. D) Reconstructed dipole time courses using the OPM (black) and SQUID (red) data.

Fig. 5 shows source localisation of the evoked (N20) response. Fig. 5A shows the averaged time courses across trials from all 13 OPM ‘channels’ (in black) and 271 SQUIDs (in red). As expected, a bipolar response is observed with the positive and negative measurements reflecting the direction of the magnetic field (out of the head and into the head respectively). Left and right panels of Fig. 5B show the spatial topography of magnetic field at the scalp level, across the 271 SQUID sensors and the 13 OPM head-cast locations, respectively. Both measured (bottom) and modelled (top) field maps are shown. The scatter plot in the centre panel shows the correlation between the modelled and measured fields for both OPMs (in black) and SQUIDs (in red). The degree of correlation for the OPMs is compelling, particularly given that the 13 ‘channels’ were measured independently using only a single sensor and 13 separate experiments. Fig. 5C shows two separate views of the N20 evoked response localisation, overlaid onto the subject-specific (MRI-extracted) cortical mesh. The motor strip has been highlighted in green and the sensory strip in blue. The location derived from OPMs is in black and the location derived from SQUIDs is in red. The coordinates and orientations of both dipole locations are shown in Table 1. Note that both dipoles localise to the right primary somatosensory cortex (S1) as would be expected. One should note here that these are source estimates based on different sensor types and for the OPMs these data represent the concatenation of 13 sequentially recorded runs, which were subsequently treated as being simultaneously acquired i.e. the OPM data are contaminated with between session physiological variability.

**Table 1:** **Source localisation of the evoked response.** Coordinates (in mm) and orientation of the evoked response location in MNI coordinates for the OPMs and SQUIDs.

	**Coordinates (mm)**			**Orientation**		
	**x**	**y**	**z**	**x**	**y**	**z**
**OPMs**	45.21	−23.44	42.89	0.73	0.68	0.11
**SQUIDs**	34.76	−25.22	35.40	0.98	−0.19	0.01

This evidence shows clearly that OPMs (even collected sequentially in the absence of a multi-channel system) can be employed to derive images of the spatial distribution of electrophysiological changes in the brain. Fig. 5D shows source-space reconstructed dipole time courses, generated independently using OPMs (black) and SQUIDs (red). The shading represents standard error across trials. Note the clear similarity in both morphology and amplitude. Note also that the standard error over trials (itself a measure of noise) is generally smaller in the OPMs compared to the SQUIDs.

Source-space SNR was estimated as described above for the evoked response. OPM SNR was estimated as 42.52 and 26.99 using the windowed and anti-averaging methods respectively. Similarly the SQUID evoked response SNR was estimated as 21.41 and 19.79 using the equivalent two methods. These measures show an improvement of 1.99 and 1.36 in evoked response SNR of OPMs compared to SQUIDs, depending on the measurement method used (however, see also Supplementary information where we show that, using optimised SQUID selection, SNR values of OPMs and SQUIDs are more comparable).

## Discussion

Significant advances in quantum sensing have led to the development of magnetic field detectors, whose external surfaces are at room temperature during operation. These sensors are able to achieve sensitivity similar to that of cryogenically-cooled devices (SQUIDs). This means that room temperature MEG, with a greatly increased flexibility of sensor placement, higher SNR, and potentially lower cost, can now be considered. Here we have employed a single commercially available OPM to highlight the utility and potential of OPM devices to drive a step change in the capabilities of MEG. We have shown that an appropriately configured OPM has a source-space SNR comparable to current SQUID systems. Previous work suggests that the effective use of an OPM system in generating accurate 3D depictions of the changes in brain current depends on both cancellation of interference and accurate knowledge of sensor placement relative to the cortex. Here we have shown that synthetic gradiometry represents a useful means to cancel interference, whilst the use of 3D printing allowed us to accurately locate the sensor on the scalp surface, with a known position and orientation relative to brain anatomy.

### Synthetic gradiometry

The fabrication of a reference array, located sufficiently far from the head to be insensitive to the neuromagnetic field, but sufficiently close to pick up the same environmental interference, has been a strategy for reduction of interference since the earliest multi-channel SQUID devices were introduced (Vrba, 2002, Vrba et al., 1991, Vrba and Robinson, 2002, Vrba and Robinson, 2001). Indeed this is the basis behind the synthetic third-order gradiometer approaches used in many commercially available instruments. Here, we employ a similar procedure, using our SQUID system to remove interference recorded at the OPM. The method we use is based on a simple regression of the reference signal. Although this was effective (in empty room data, 97% of variance in the OPM signal was explained and removed by our technique) we stress that this represents only a simple demonstration and it relies on all interference fields having the same spatial signature. For example, consider a situation in which two separate interference sources exist, but with different spatial variation across the MSR: Interference source A has a greater magnitude at the OPM compared to the reference array, whereas interference source B has greater magnitude at the reference array compared to the OPM. In our simple regression formulation, a single set of weights is used to map the reference to the OPM, meaning that a single mapping would have to account for both sources. This means that the prediction of source A interference at the OPM, based on the reference would likely be an under-estimate, and similarly the prediction of source B interference would be an over-estimate. In other words, no single set of weights can account for both sources. In reality, the mapping will be best ‘tuned’ to the largest source of interference, and this is why, in Fig. 3A where a single mapping is used across all frequencies, the 50 Hz mains artefact is well suppressed, but little effect is noted at other frequencies. Here, this problem was ameliorated somewhat by assuming that separate interference sources manifest within different frequency bands; using regression coefficients derived independently for each band, we showed that a greater efficiency of interference cancellation results (Fig. 3B). However, if more than one interference source exists within a single band, then the same problem results. Importantly, a significant limitation of the approach taken here is that the CTF SQUID array covers a relatively small spatial volume within the MSR – meaning that the interference magnitude is approximately constant for all channels (i.e. the spatial variation of interference is poorly sampled). This is a consequence of using SQUID sensors since all sensors must be kept within the liquid helium dewar. If we were to employ non-cryogenic devices as references, no such limitation would apply and the spatial variation of the interference inside the MSR could be better characterised and linear (or even non-linear) mappings to the OPM array at the scalp would yield more efficient interference cancellation. Further, extra channels could even be placed close to common sources of biological interference (e.g. the heart) such that interference from these sources could also be more efficiently removed. This, coupled with the band-specific approaches demonstrated here, we speculate, would facilitate improved efficiency of synthetic gradiometer interference cancellation.

### Head-cast technology

One of the most important factors in our experimental set-up was the use of 3D printing to specify, a priori, the configuration of sensors and to stabilise those sensors (relative to the head and the MSR) during data collection. 3D printing is becoming commonplace for fabrication of structures with unusual geometries. Here, we based the head-cast print on the surface of the subject's scalp extracted from their anatomical MRI image. We then placed the sensor locations above the right somatosensory cortex to optimally capture the maximum and minimum of the magnetic field produced by the N20 response to median nerve stimulation. As such, the head-cast we used was designed to target a specific region of interest, facilitating high specificity with relatively few (13) sensor locations. It is noteworthy that this same idea could be used to target any cortical (or subcortical) region, as long as one knows the location and orientation of the interesting segment of cortical surface, information which can be obtained from anatomical MRI. Equally important here was the fact that the 3D-printed head-cast meant that the location of the sensitive volume of each sensor, and its sensitive orientation relative to the brain anatomy was known accurately. This information is critical if one is to perform accurate localisation of the neuronal generators of the recorded neuromagnetic signals (and becomes all the more important now the relative sensor locations are no longer fixed by cryogenics). Several papers have studied the influence of inaccurate forward modelling to beamformer localisation performance in MEG (Hillebrand and Barnes, 2003) and EEG (Dalal et al., 2014, Steinsträter et al., 2010); showing that co-registration and segmentation errors, insufficient sensor coverage and inaccuracies of volume conductor models can compromise beamformer localisation accuracy, yielding errors of up to several centimetres. As SNR of the data is increased, accurate forward modelling becomes of greater importance. Our previous work (Boto et al., 2016) has shown that, with increasing error on the forward model, (beamformer) inverse solutions fail faster in the presence of high SNR OPM data compared to lower SNR SQUID data. It is for this reason that the 3D printing technology used here is a critical step to ensure a well characterised detector array. The approach taken, which involves an individual MRI followed by bespoke printing of a subject-specific head-cast, may seem expensive and complex, however it removes a number of potential of sources of modelling error. This is particularly important as we set out to characterize the sensitivity and cross-talk between these new sensors but one would hope with time, the positioning requirements will become less stringent as the sensors become more completely characterized (López et al., 2012, Martínez-Vargas et al., 2016). The future use of flexible (EEG type) caps and an optical system for sensor location and orientation measurements may also become viable.

### A comparison of OPM and SQUID: signal magnitude and SNR

One of the principal advantages of OPMs over SQUIDs is the reduced distance between cortical source(s) and sensor. Because neuro-electrical sources are dipolar, the fields that they generate follow an inverse square law, giving rise to a non-linear increase in signal magnitude as the sensors move closer to the skull. This means that the magnitude of the measured signal using an OPM is larger than that recorded using a SQUID. This comparison is shown in Fig. 4, which shows an approximate fourfold increase in signal magnitude for evoked and induced responses. However, this comparison requires explanation: first, the degree of improvement afforded by the OPM is a function of the location of the source of interest in the brain. This is shown by the calculation in our Appendix A. The non-linear nature of the inverse square law means that, although moving detectors closer to the scalp will offer an increase in signal magnitude for *all* brain regions, the improvement for shallow sources will be larger than for deep sources. More specifically, whilst improvements in the cortex (shallow sources) could be as high as eightfold, improvements for deeper sources (e.g. thalamus) may be closer to twofold. This means that our observed increases in signal magnitude, whilst typical for the cortex, will not be achievable across the whole brain. Second, here we compare the magnitudes of effects measured using a magnetometer (OPM) and gradiometer (SQUID). The gradiometer is hardwired and comprises two coils, oriented radially with a 5 cm baseline. Both coils will pick up the neuromagnetic field and the subtraction of the signal measured at the coil farthest from the head, from that closest to the head, will mean a slight reduction of the response magnitude compared to what would be measured using a SQUID magnetometer (in which only the closest coil would be used). In this case we estimate that a SQUID magnetometer at the position of the closest gradiometer coil would likely increase the signal by approximately 36% (based on nearest sensor to source distance of 53 mm). In summary, our results confirm that OPMs offer increased signal magnitude compared to SQUIDs. However, this advantage is inhomogeneous with respect to location, and is tempered marginally by gradiometer measures.

For the above reasons we were motivated to compare SNR of OPMs and SQUIDs in source space. Such a comparison takes into account both our ability to reduce environmental interference (e.g. via gradiometry) and our ability to suppress brain sources of no interest from modelled time courses (itself a function of sensor-space SNR). When comparing 271 SQUIDs to 13 OPMs, we found marginally less variability in the source level evoked response over trials when using the OPMs, even though the OPM data were recorded over multiple sessions (i.e. had an extra source of physiological noise). Our quantitative metrics agreed in showing marginally better SNR for OPMs. However, these metrics compared 271 SQUID locations to 13 OPM locations. We were therefore motivated to reduce the SQUID sensor count to 13. When comparing 13 OPMs to 13 (optimally selected) SQUIDs, we found SNR values to be more comparable (see Supplementary information). This said, we stress that these measurements were made in a single subject. Furthermore, OPM measurements were sequential whilst SQUID measurements were simultaneous. This means that the effects of habituation (see e.g. Christoffersen (1997)) over trials and multiple experiments cannot be discounted. We therefore look to future studies with multi-channel OPM arrays to allow better characterisation of source-space SNR and spatial resolution.

### Perspective

At the time of writing, OPMs are a nascent technology and there is considerable room for improvement (especially in terms of sensitivity). For example, in our measurements, the coils mounted on the OPM were used to cancel a significant DC offset field (stray Earth's field) inside the MSR. Although internal coils were sufficient to deal with this, inhomogeneities in this compensation could reduce the sensitivity of the OPM (as well as marginally change the effective gain). With this and other similar considerations in mind, the fact that MEG data acquired using an OPM are comparable in quality to that acquired using established SQUID systems (with their many decades of development and optimisation) is encouraging. The OPMs are fully-integrated with a low-power laser inside the sensor, the technology is therefore robust and relatively maintenance free. More importantly, the flexibility to place sensors anywhere on the scalp surface not only increases signal magnitude but further increases the patient populations that can be studied, and the types of paradigms that can be employed. The most obvious potential benefit of this flexibility is in the imaging of infants where current ‘one size fits all’ fixed array systems simply do not get close enough to the head to measure meaningful signals (e.g. in a baby, the increase in signal magnitude produced by using OPMs would be more dramatic). In addition, this flexible sensor placement will also allow better coverage of different regions, for example allowing measurement of electrical activity in the cerebellum, or even the spinal cord. Further, OPM array geometry can be optimised via modelling (similar to that undertaken here) to target deeper brain structures such as the thalamus or hippocampus. Such flexible determination of the sensor array, coupled with flexible positioning of reference sensors (see above) is a significant consideration in the argument to replace SQUIDs with OPMs. That said, significant engineering challenges still remain: for example, the operation of a multi-channel array is complicated by cross-talk between sensors from both the internal field zeroing coils, and from field modulation used for lock-in detection. These interfering fields between sensors in close proximity would significantly alter both the sensor gain, and potentially the orientation of the sensitive axis of detection.

We note that this is an exciting time for MEG with several types of new sensor now in development. In addition to OPMs, these include new devices such as the HyQuid™ (Shelly et al., 2016) and nitrogen-vacancy magnetometers (Taylor et al., 2008) which offer the potential of optically imaging magnetic field changes in the brain.

## Conclusion

Thanks to the commercialization of single-unit OPMs, it is now possible to create flexible multi-channel arrays for detection of the human magnetoencephalogram. In this paper, we have presented MEG recordings made with a single OPM in combination with a 3D-printed head-cast designed to accurately locate and orient the sensor. Because OPMs are configured as magnetometers (as distinct from gradiometers) they are highly sensitive to environmental interference. However, we have shown that this problem can be ameliorated via the use of simultaneous reference sensor recordings. Using median nerve stimulation, we showed that the OPM can detect both evoked and induced changes when placed over sensory cortex; with signals exhibiting ~4 times the magnitude of equivalent metrics made using SQUIDs. Finally, we have shown that our high SNR OPM measurements, coupled with high accuracy in OPM placement (afforded by the head-cast) allow accurate localisation of the evoked response to somatosensory cortex. Our results highlight the opportunity to generate low cost OPM-based MEG systems, which may offer a step change in sensitivity compared to the current generation MEG instruments.

## Conflict of interest

Co-authors Vishal Shah, Svenja Knappe and Orang Alem are directors at QuSpin – a commercial entity selling OPM magnetometers. They built the OPM device used here and advised on its operation but played no part in the subsequent measurement or data analysis. Mark Lim is the director of Chalk Studios Ltd., a graphic design company which designed and built the nylon head-cast. The remaining ten authors are academic scientists who have no commercial interests.

## Figures and Tables

**Fig. A1:**
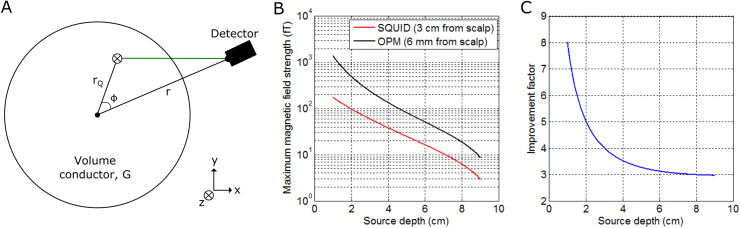
**Assessing signal magnitude as a function of source depth:** A) Schematic of a simple analytical model. B) Magnitude of the measured radial magnetic field as a function of source depth for SQUID (red) and OPM (black). C) The measured improvement (ratio of fields measured by OPM and SQUID) as a function of depth.

## Appendix A Relative advantages for shallow and deep sources

The improvement in the magnitude of the measured MEG signal afforded by moving a detector closer to the head is dependent on the location in the head of the source generating the signal. To show this dependence, a simple analytical calculation can be undertaken. Consider the system in Fig. A1A where a current source is located at position **r**_Q_ inside a volume conductor, G, and a detector is located at position **r** outside G. The magnetic field, **B**, at **r** is given by the Geselowitz formula:

(A1) Br=μ04π∫GJPrQ×r-rQ‖r-rQ‖3dv'-μ0σ4π∫SVRnR×r-R‖r-R‖3ds

Here, **J**^P^ denotes primary current flow and thus the first term, which represents a volume integral over G, computes the magnetic field arising from primary (dendritic) current flow. In the second term, which represents an integral over the surface, S that bounds the conductor, G, V denotes electrical potential, σ represents conductivity inside the conductor, **R** is any point on the surface, S and **n**(**R**) is a unit vector normal to S at **R.** The second term describes the contribution of volume currents to the magnetic field at **r**. To simplify the calculation, we assume that G is spherical. Further, we assume that only the radial component of field is measured (which is the case for the experiments carried out here so that the measured field, B_r_=**B**(**r**)·**e**_r_(**r**) where **e**_r_(**r**) is a unit radial vector at **r**. In this case, since **n** is parallel to **R** and the radially oriented detector means that **e**_r_ must be parallel to **r**, the volume current term is reduced to zero and measured field is approximated as

(A2) Brr=μ04π∫G[JPr'×r−rQ]·err‖r−rQ‖3dv'

If we further assume that the primary current, **J**^P^, is a current dipole, so that **J**^P^=**Q** δ(**r′**−**r**_Q_) (where δis the Dirac delta function and **Q** represents the magnitude and direction of the dipole). The measured radial field then reduces to

(A3) $$B_{r}\left(r\right)=-\frac{\mu_{0}\left(Q\times r_{Q}\right)\cdot e_{r}}{4\pi {\left\|r-r_{Q}\right\|}^{3}}$$

Considering a dipole oriented normal to the radial direction (specifically, the z-direction – see Fig. A2A) and (without loss of generality) limiting **r** and **r**_Q_ to a single plane, Eq. (A3) reduces further to

(A4) $$B_{r}\left(r\right)=-\frac{\mu_{0}\left|Q\right|\left|r_{Q}\right|\sin(\phi )}{4\pi {\left({\left|r\right|}^{2}+{\left|r_{Q}\right|}^{2}-2\left|r\right|\left|r_{Q}\right|\cos\left(\phi \right)\right)}^{3/2}}$$

This simple expression allows an approximation of measured field, for different scalp-detector distances (which can be set using |**r**|) and different source depths (which can be set using |**r**_Q_|). The expression in Eq. (A4) is asymmetric with respect to the angle φ (between **r** and **r**_Q_) and the angle at which the maximum field is found varies as a function of both |**r**| and |**r**_Q_|. Specifically, the optimal angle at which a sensor is placed is found by differentiating Eq. (A4) so that

(A5) $$\cos\left(\phi \right)=\frac{-\left({\left|r\right|}^{2}{+\left|r_{Q}\right|}^{2}\right)+\sqrt{{\left|r\right|}^{4}+{\left|r_{Q}\right|}^{4}+14{\left|r\right|}^{2}{\left|r_{Q}\right|}^{2}}}{2\left|r\right|\left|r_{Q}\right|}$$

In order to assess the improvement in signal magnitude afforded by OPMs, as a function of dipole depth in the head, Eqs. (A4) and (A5) were used. We assumed that the head was spherical with radius 10 cm. A superconducting gradiometer (5 cm baseline) was placed 3 cm from the scalp surface (i.e. a gradiometer was simulated by computing B_r_ twice, with |**r**|=13 cm and |**r**|=18 cm, and subtracting the results). An OPM (magnetometer) was placed 6 mm from the scalp surface (i.e. |**r**|=10.6 cm). A source magnitude of |**Q**|=10 nA·m was used and the dipole depth was varied by changing |**r**_Q_| between 1 cm and 9 cm. The angle at which the detector was placed, φ, was defined independently for all |**r**_Q_| by Eq. (A5), and was larger for deeper sources as expected. Results are shown in Fig. A1B and A1C.

Fig. A1B shows the analytically calculated magnitude of the magnetic field, for an OPM (black) and a superconducting coil (red), as a function of source depth in the brain (source depth=10−|**r**_Q_| cm). Note that the OPM offers an improvement in source magnitude for sources anywhere in the brain, however the field magnitude falls off faster with increasing depth in the OPM compared to the SQUID gradiometer. This means a greater amplification of nearby sources compared to distal sources when using an OPM detector. This effect, which is a consequence of the inverse square nature of Eq. (A1), is further demonstrated by Fig. A1C which shows the improvement afforded by an OPM compared to a SQUID gradiometer as a function of depth. Note that improvement is calculated as the ratio of fields measured by OPM and SQUID gradiometer (i.e. a value of 5 means the OPM signal is 5 times that of the SQUID). For shallow sources, an 8-fold improvement in signal magnitude is predicted; however this improvement falls to only a 3-fold increase when considering sources close to the centre of the brain. This observation has important consequences for understanding SNR differences between the two systems. In the present paper, in both the SQUID and OPM systems, (following gradiometer correction) signal from the brain dominates environmental interference and so the challenge of enhancing SNR becomes more about supressing ‘brain noise’ rather than environmental noise. For relatively shallow (cortical) sources like the somatosensory source in our experimental results, the more rapid fall off of signal with depth gives the OPM a natural advantage compared to the SQUID.
