# Supplementary material for: A new generation of magnetoencephalography: Room temperature measurements using optically-pumped magnetometers
Source: Neuroimage. 2017 Apr 1;149:404–14. doi: 10.1016/j.neuroimage.2017.01.034 (PMC5562927; doi:10.1016/j.neuroimage.2017.01.034)
Supplement: Supplementary file 1 — Supplementary material [file mmc1.pdf]

A new generation of magnetoencephalography: Room temperature measurements using optically-pumped magnetometers

Elena Boto, Sofie Meyer, Vishal Shah, Orang Alem, Svenja Knappe, Peter Kruger, Mark Fromhold, Mark Lim, Paul Glover, Peter Morris, Richard Bowtell, Gareth Barnes and Matthew Brookes

Supplementary information

Motivation: In the main manuscript we assessed the source-space SNR of the evoked response, comparing results from SQUID and OPM measurements. To do this, we used a dipole fitting algorithm to estimate the location and time course of the generator of the evoked response in somatosensory cortex. However, for the SQUID system, the dipole fit was undertaken using signals from 271 SQUID-based channels. In contrast, the equivalent fit for the OPM set-up used 13 measurement locations. Such a comparison is potentially problematic: the increased number of channels in the SQUID array may lead to a better fit and hence a more accurate reconstructed time course. However conversely, the positioning of the OPM sensors was optimised, whereas the SQUID array had whole brain coverage. It is apparent from Fig. 5B that differences between modelled and measured SQUID measurements are driven by activity in the left hemisphere, which cannot be explained by our single dipole solution in the right hemisphere. It is therefore conceivable that the restricted OPM coverage, optimised to track the dominant right hemispheric activity and thus unaffected by simultaneous activity in regions with no sensor coverage, may be advantageous. To address this confound, we undertook further analyses, where the number of SQUID sensors was reduced to 13.

Method: Our reduced SQUID computation was achieved in two ways: first, we chose 13 sensors closest to the midpoint of the field map extrema (the midpoint method), and used the dipole fitting procedure as described in the main manuscript. Second (the optimised method), we took the location and orientation of the dipole fit to the 271 sensors and calculated its time series via the maximum a posteriori estimate (Dale et al., 2000), based on the 13 SQUID channels of largest magnitude at 20 ms. For both methods, SNR was measured in two ways (as in the main manuscript): the signal was calculated as the peak-to-peak change. In the first case, noise was estimated as the standard deviation in the window 0.6-1.5 s post stimulus. In the second case, noise corresponded to the standard deviation over the whole trial after an anti-averaging procedure.

Results: Fig. S1 shows a comparison of source localisation of the evoked (N20) response using 13 OPMs (recorded sequentially) and 13 SQUIDs. The left and right panels of Fig. S1A show the spatial topography of magnetic field at the scalp level, across 13 SQUID sensors (midpoint method) and OPM locations, respectively. Both measured (bot-

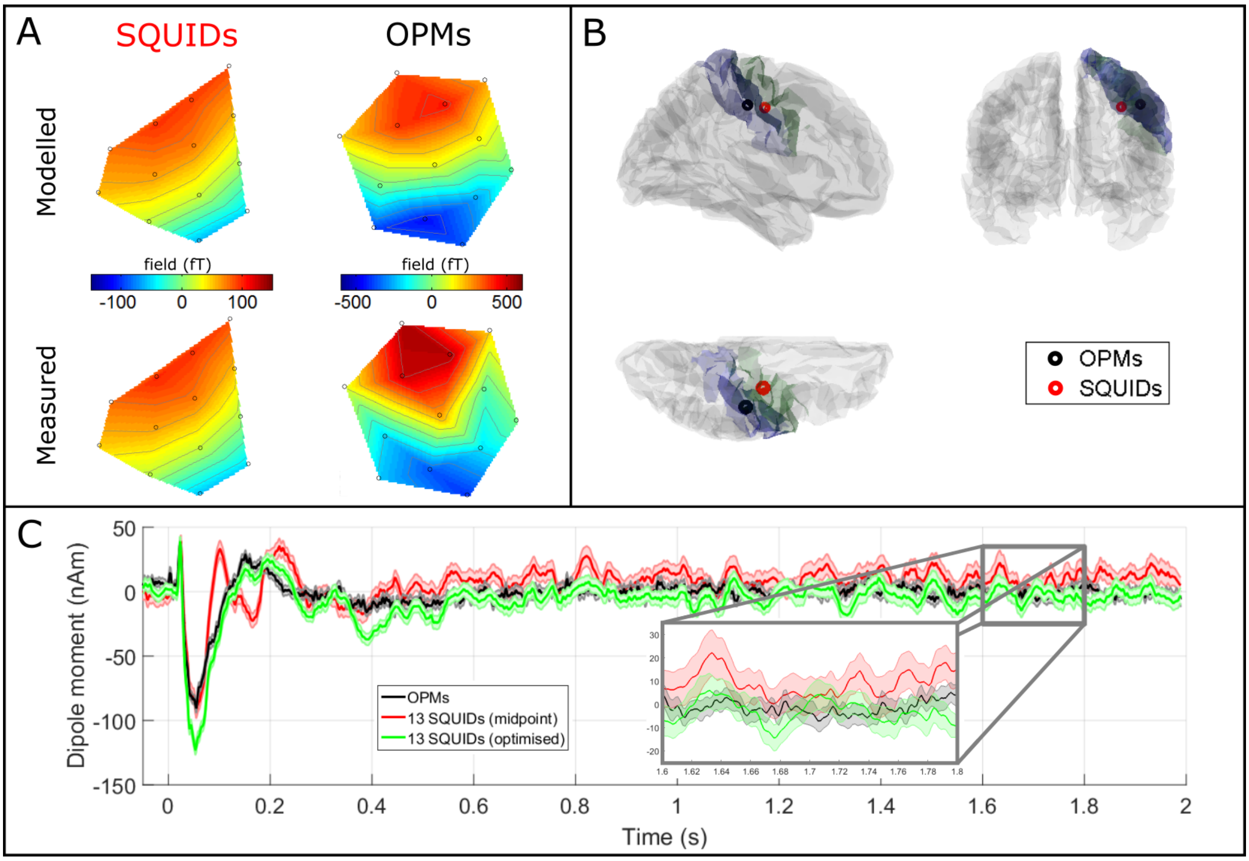

Fig. S1. Source localisation of the evoked response. A) Spatial topography of magnetic field recorded using either 13 SQUID sensors (left) or 13 OPM sensors (right). The upper panel shows the modelled fields and the lower panel shows the measured fields. B) N20 evoked response localisation overlaid onto the cortical mesh. C) Source-space reconstructed dipole time courses, generated independently using 13 OPMs (black), 13 SQUIDs (midpoint method, in red) and 13 SQUIDs (optimised method, in green).

tom) and modelled (top) field maps are shown and are in good agreement. Fig. S1B shows three separate views of the N20 evoked response localisation, overlaid onto the subject-specific (MRI-extracted) cortical mesh. The motor strip is highlighted in green and the sensory strip in blue. The location derived from OPMs is in black and the location derived from 13 SQUIDs (midpoint method) is in red. Note that the location and orientation of the dipole for the optimised method is identical to that for 271 channels, shown in the main manuscript. Finally, Fig. S1C shows source-space reconstructed dipole time courses, generated independently using 13 OPMs (black), 13 SQUIDs using the midpoint method (red), and 13 SQUIDs using the optimised method (green). The shading represents the standard errors across trials. These measures show that using our midpoint method, OPMs still show an improvement in SNR of the evoked response over SQUIDs. However, using the optimised method, SNR values are more comparable (see Table S1).

Discussion: Overall, the dipole fit location, time course and quantitative values of SNR derived using a reduced SQUID channel count, were similar to those derived when

Table S1
SNR values related to the evoked response.

	SNR	
	**windowed method**	**anti-averaging method**
**OPMs**	42.52	26.99
**271 SQUIDs**	21.41	19.79
**13 SQUIDs** (midpoint method)	22.21	17.60
**13 SQUIDs** (optimised method)	27.94	33.86

including all 271 sensors. This shows that any artefact in reconstruction, caused by left hemispheric activity in the 271 channel measurement is having little overall effect. In agreement with our main manuscript, the quantitative values of SNR show OPMs to be comparable to SQUIDs. However, we stress that these values can only be considered representative and should not be over-interpreted, since they result from measurements on a single subject and are based on comparing measurements formed by the spatial concatenation of 13 sequentially recorded runs for OPMs, with a single run using simultaneous acquisition across the SQUID sensors. Effects of habituation cannot therefore be ruled out and we look to future studies with multi-channel OPM arrays to allow better characterisation of source-space SNR and spatial resolution.

Dale, A. M., Liu, A. K., Fischl, B. R., Buckner, R. L., Belliveau, J. W., Lewine, J. D., and Halgren, E. (2000). Dynamic statistical parametric mapping: combining fMRI and MEG for high-resolution imaging of cortical activity. *Neuron*, 26(1):55–67.
